# Exogenous SNP Alleviates Drought Stress in Wheat During the Grain-Filling Stage by Modulating *TaP5CS* Gene Transcription

**DOI:** 10.3390/ijms26020618

**Published:** 2025-01-13

**Authors:** Xinyu Xue, Ruqing Li, Menghan Zhang, Sixu Jin, Haifang Jiang, Chongju Wang, Yifei Pang, Ruili Xue, Yuexia Wang

**Affiliations:** College of Life Sciences, Henan Agricultural University, Zhengzhou 450046, China

**Keywords:** wheat, drought stress, sodium nitroprusside (SNP), pyrroline-5-carboxylate synthase (*TaP5CS*), yield traits

## Abstract

Drought stress severely damages wheat growth and photosynthesis, and plants at the grain-filling stage are the most sensitive to drought throughout the entire period of development. Exogenous spraying of sodium nitroprusside (SNP) can alleviate the damage to wheat caused by drought stress, but the mechanism regulating the proline pathway remains unknown. Two wheat cultivars, drought-sensitive Zhoumai 18 and drought-tolerant Zhengmai 1860, were used as materials when the plants were cultivated to the grain-filling stage. The results show that under drought stress, SNP pretreatment effectively improved the physiological basis of photosynthesis and water use efficiency of the two cultivars, increased their tolerance to photosystem II (PSII) damage, and maintained a normal photosynthetic rate and yield. Drought stress induced an increase in pyrroline-5-carboxylate synthase (*TaP5CS*) gene transcription, and a comparatively greater increase was detected in Zhengmai 1860. When SNP treatment was applied before drought exposure, *TaP5CS* transcription was further enhanced. Induction of *TaP5CS* transcription promoted proline accumulation in response to drought stress, increased osmotic ability, and maintained the net photosynthetic rate, thereby increasing the accumulation of dry matter and yield traits. In this study, exogenous SNP regulates the transcription of genes related to the proline metabolism pathway and provides a theoretical basis for the establishment of wheat cultivation technology using SNP to resist drought stress.

## 1. Introduction

As one of the main food crops in the world, wheat accounts for approximately 30% of the global population and is the second most commonly grown crop in the world [1]. Drought stress damages the physiological mechanisms, photosynthesis, and dry matter production of wheat and has become an increasing threat that severely reduces wheat yield because of global climate change [2,3]. During the grain-filling stage, osmotic stress caused by drought limits nutrient uptake and reduces the metabolic rate, which is most serious for yield reduction when plants are subjected to drought stress throughout the entire growth period [4].

Environmental stress is sensed and transduced through a cascade of signaling molecules, which ultimately regulate the elements of stress-responsive genes. This regulation initiates the synthesis of various proteins, including transcription factors (TFs), enzymes, molecular chaperones, ion channels, and transport proteins, or modulates their activity [5]. Transcription factors have received a lot of attention this year, among TFs, WRKY, CBF, MYB, bHLH, ERF, and MYB genes have received much attention in the past decade. The *MbWRKY1* gene, isolated from Malus baccata, has been shown to enhance drought tolerance in transgenic tobacco plants following its introduction [6]. Transgenic plants overexpressing the *MbWRKY4* gene exhibit an enhanced oxidative stress response under high-salinity conditions, highlighting the positive role of *MbWRKY4* in mediating salt stress tolerance [7]. Moreover, the *MbWRKY46* gene has been shown to enhance the expression of downstream stress-responsive genes in Arabidopsis thaliana (*AtKIN1*, *AtRD29A*, *AtCOR47A*, *AtDREB2A*, *AtERD10*, and *AtRD29B*) under cold and drought conditions, thereby improving the plant’s tolerance to these stresses [8]. Furthermore, analysis of the expression levels of cold- and drought-stress-related genes in Arabidopsis thaliana transgenic lines overexpressing *MbWRKY53* suggests that this gene regulates the expression of stress-responsive genes through multiple pathways, including the CBF, SOS, Pro synthesis, and ABA-dependent pathways, thereby enhancing the transgenic Arabidopsis’s adaptability to cold and drought environments [9]. The CBF transcription factor is a crucial regulator of plant stress tolerance, playing an indispensable role in plant resistance to adverse environmental conditions. The *MbCBF2* gene positively regulates the expression of key downstream genes associated with cold stress, such as *AtCOR15a*, *AtERD10*, *AtRD29a/b*, and *AtCOR6.6/47*, under low-temperature conditions. Additionally, it positively regulates the expression of key genes involved in salt stress, including *AtNCED3*, *AtCAT1*, *AtP5CS*, *AtPIF1/4*, and *AtSnRK2.4*. Overexpression of *MbCBF2* thus further enhances the adaptability and tolerance of transgenic plants to both low-temperature and high-salinity environments [10]. MYB transcription factors regulate the biosynthesis of anthocyanins. And bHLH factors play a significant role in plant resistance to drought, cold, and salt stresses [11]. Studies have shown that overexpression of the foreign gene *StMYBA1* in tobacco stimulates the expression of endogenous bHLH partners. The increased expression of these bHLH partners is crucial for anthocyanin production in plant tissues. *LcbHLH1* and *LcbHLH3* are key partners involved in the regulation of anthocyanin biosynthesis in tobacco and lychee by *LcMYB1* [12]. In addition, the expression pattern of *XsAP2/ERF* transcription factors under low-temperature treatment further confirms their significant role in the response to abiotic stress [13].

In addition to transcription factors, which play a crucial role in plant resistance to abiotic stress, several enzymes also contribute to stress tolerance. The results of relevant studies have shown that when wheat is subjected to drought stress, the proline content significantly increases, and its increasing levels in different cultivars may be associated with the drought tolerance of specific cultivars [14]. As an osmoprotectant that stabilizes cell membranes, proline can improve the protection of antioxidant enzymes, thereby scavenging free radicals, maintaining and improving water storage and use status, and reducing water loss under drought stress [15,16]. The drought tolerance of wheat can be increased by both exogenous proline treatment [17] and experimentally genetic engineering to promote endogenous proline synthesis [18]. The accumulation of a large amount of proline regulates osmosis, increases membrane stability to reduce water loss, and then maintains normal turgor pressure, which can effectively relieve the damage to plants caused by drought stress [17,18].

Pyrroline-5-carboxylate synthase (P5CS) is a dual-function enzyme that converts glutamate to pyrroline-5-carboxylate during the glutamate synthesis pathway of proline; thus, P5CS is a key enzyme for proline biosynthesis in response to stressful environments [19]. The overexpression of P5CS has already been shown to improve drought tolerance by increasing proline accumulation in grapevine [20] and wheat [21]. When wheat plants are subjected to drought stress, *TaP5CS* gene transcription is significantly upregulated by foliar zinc application, which can effectively relieve damage and improve grain yield [22]. A previous study revealed that the gene encoding the wheat *TaP5CS* gene (accession no. *TraesCS1D02G280700*) was significantly upregulated in response to drought stress and was positively associated with cultivar-dependent stress tolerance [23]. However, the regulatory mechanism of the wheat *TaP5CS* gene has not been fully elucidated. Pyrroline-5-carboxylate reductase (P5CR) is an essential housekeeping enzyme widely present in both prokaryotic and eukaryotic organisms. Under the action of NAD(P)H, pyrroline-5-carboxylate (P5C) is converted into proline through the catalytic activity of P5CR [24]. Proline dehydrogenase (ProDH) is a key enzyme located in the mitochondria that catalyzes the degradation of proline. Reducing ProDH activity is important for regulating osmotic balance, preventing osmotic stress-induced damage in plants, scavenging free radicals, and protecting cell structures [25]. P5CS, P5CR and ProDH together constitute the proline synthesis and metabolism pathway. SAMDC is a key enzyme in the plant polyamine synthesis pathway. It catalyzes S-adenosine methionine (SAM) to form decarboxylated SAM, providing aminopropyl donors for polyamine biosynthesis [25]. Polyamines play an important role in the plant response to stress, regulating physiological processes and enhancing plant stress tolerance [26].

Nitric oxide (NO) plays an important role in plant responses to various abiotic stresses, helping to promote growth and development [27]. Drought stress leads to a large accumulation of reactive oxygen species (ROS), which damage the cell membrane system, whereas NO alleviates the lipid peroxidation caused by osmotic stress [28,29]. Sodium nitroprusside (SNP) is an NO donor that plays an important role in regulating physiological and biochemical processes, such as photosynthesis and stomatal conductivity, in response to abiotic stress [30]. Previous reports have shown that exogenous SNP spray can effectively improve antioxidant activity, scavenge excessive ROS, reduce damage caused by oxidative stress, and maintain photosynthesis by increasing chlorophyll synthesis in plant leaves, thus increasing dry matter accumulation and crop yield [31]. Under lead stress, SNP improved the accumulation of proline, restored the performance of photosynthetic organs, and promoted the growth of rice seedlings [32]. However, no reports have revealed the association between the exogenous SNP-mediated regulation of proline accumulation and the mitigation of drought stress damage on wheat yield.

This study utilized two wheat cultivars with different drought tolerance levels, Zhoumai 18 and Zhengmai 1860, as experimental materials. Exogenous SNP was applied through foliar application during the grain-filling stage to assess its potential physiological and molecular effects on the response to drought stress. The expression patterns of the *TaP5CS* gene were examined during proline accumulation. These findings provide a foundation for developing effective antidrought cultivation strategies for wheat during the grain-filling stage.

## 2. Results

### 2.1. Effects of Exogenous SNP on Phenotype and Physiological Characters of Flag Leaves of Wheat Under Drought Stress

**Figure 1:**
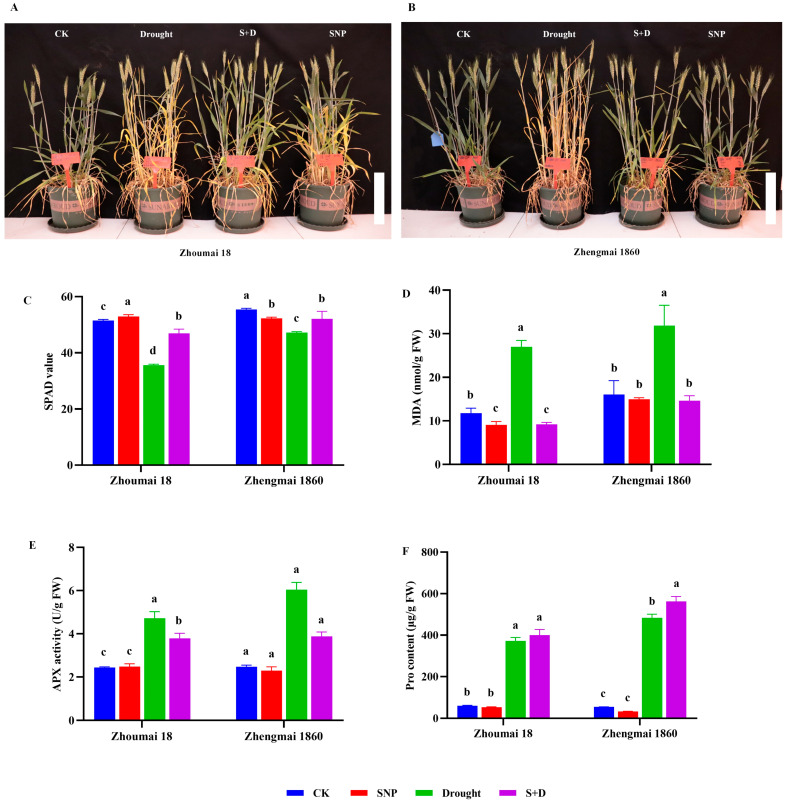
Phenotypes (**A**,**B**), SPAD (**C**), MDA content (**D**), APX activity (**E**), and Pro content (**F**) of Zhengmai 1860 and Zhoumai 18 under drought stress and SNP pretreatment at the grain-filling stage. The wheat plants were cultivated to the grain-filling stage and then subjected to drought stress. The SNP and S+D groups were sprayed with 100 mg/L SNP for 7 days. The watering of the plants in the drought and S+D groups was subsequently stopped to simulate drought stress, whereas those in the CK and SNP groups were watered normally. The data are presented as the means ± standard deviations of three replicates. Different letters indicate significant differences between all treatments at the level of *p* < 0.05. Scale bars (**A**,**B**), 5 cm.

Under drought stress, the leaves of the two wheat cultivars displayed yellowing compared to the leaves of control plants, and this phenotypic consequence of drought stress was more obvious in Zhoumai 18 (Figure 1). Compared with those of drought-only plants, when the wheat plants were pretreated with SNP, most of the leaves appeared green under drought stress. Moreover, the color change in Zhengmai 1860 was more significant than that in Zhoumai 18, since almost all the leaves appeared green. The SPAD (Soil and Plant Analyzer Development) value positively correlated with the chlorophyll content, which was consistent with the phenotype of the plant leaves. Drought stress led to significant decreases in SPAD values (*p* < 0.05), with a more significant decrease in Zhoumai 18, which showed a 21% decrease, compared to that in the control group, as only an 8% decline was observed in Zhengmai 1860. When the plants were pretreated with SNP before drought exposure, the SPAD value significantly increased. Even the SPAD value of Zhengmai 1860 could be restored to the control level. The results show that the exogenous application of SNP resulted in relatively stable chlorophyll contents.

Malondialdehyde (MDA) is the expression of lipid peroxidation in plant cell membranes, and its content is closely related to plant aging and stress injury. When plants are subjected to drought stress, the MDA content will increase significantly. Ascorbate peroxidase (APX) is an important antioxidant enzyme in plant active oxygen metabolism; it is an especially key enzyme in chloroplasts to for H_2_O_2_ removal and is the main enzyme in vitamin C metabolism. H_2_O_2_ is a natural product of the photosynthetic electron transport chain and some enzyme reactions in plant chloroplasts, and it is a toxic reactive oxygen species. Drought stress increased the MDA content and APX activity of both wheat cultivars, but when the grain-filling plants were pretreated with exogenous SNP, both metrics decreased significantly (*p* < 0.05) (Figure 1). However, under drought stress, APX activity was still 57% and 55% greater in SNP-pretreated Zhengmai 1860 and Zhoumai 18 than in their respective normal watering controls (*p* < 0.05). The proline contents of both wheat cultivars increased significantly in response to drought stress (*p* < 0.05) (Figure 1). Compared with only a 5.2-fold increase in Zhoumai 18, the proline content in the flag leaves of Zhengmai 1860 increased by 7.8-fold under drought stress. Although exogenous SNP pretreatment had no obvious influence on the proline content when the wheat plants were watered normally, the proline content in Zhengmai 1860 further improved under drought conditions (*p* < 0.05).

**Figure 2:**
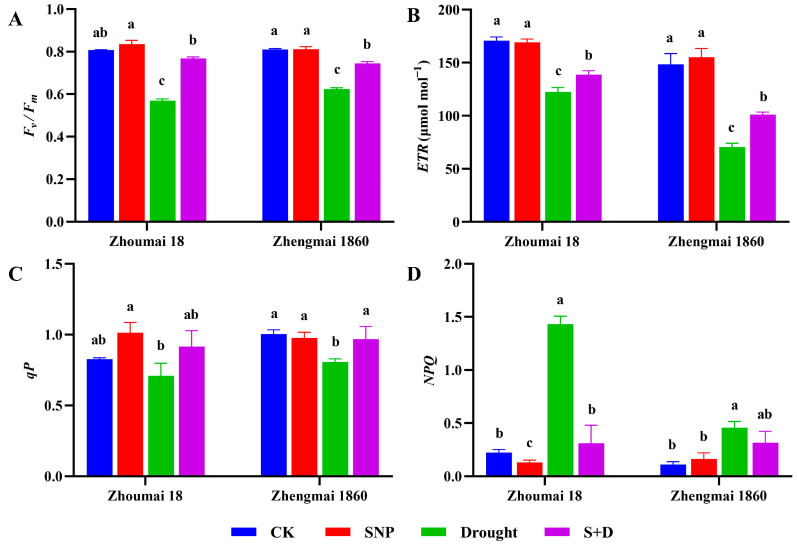
*F_v_*/*F_m_* (**A**), *ETR* (**B**), *qP* (**C**) and *NPQ* (**D**) of Zhengmai 1860 and Zhoumai 18 under drought stress and SNP pretreatment at the grain-filling stage. The wheat plants were cultivated to the grain-filling stage and then subjected to drought stress. The SNP and S+D groups were sprayed with 100 mg/L SNP for 7 days. The watering of the plants in the drought and S+D groups was subsequently stopped to simulate drought stress, whereas those in the CK and SNP groups were watered normally. The data are presented as the means ± standard deviations of three replicates. Different letters indicate significant differences between all treatments at the level of *p* < 0.05.

*F_v_*/*F_m_* value is an important parameter to measure plant photosynthetic efficiency, which reflects the maximum photochemical quantum yield of photosystem II (PSII), that is, the maximum potential photosynthetic capacity of plants after full dark adaptation. Electron transfer rate is an important parameter in plant physiology, reflecting the electron transfer rate in the chlorophyll photozyme complex during plant photosynthesis. The significance of the photochemical quenching coefficient (*qP*) is to characterize the light energy conversion efficiency of plant leaves in photosynthesis. The higher the *qP*, the smaller the non-photochemical quenching of photosynthetic pigments and the higher the photosynthetic efficiency Compared with those in the control group, under drought stress, the chlorophyll fluorescence parameters *F_v_*/*F_m_* and *ETR* decreased significantly in both wheat cultivars, while *qP* decreased slightly but not obviously in Zhoumai 18. When the plants were pretreated with exogenous SNP before drought exposure, the *F_v_*/*F_m_*, *ETR*, and *qP* values were clearly restored, and the *qP* value was restored to the level of normally watered control. Non-photochemical quenching (*NPQ*) reflects the ability of plants to dissipate excess light energy into heat, that is, the photoprotection ability of plants. Conversely, drought stress led to a significant increase in *NPQ*. Compared with Zhoumai 18, Zhengmai 1860 presented a lower increase in *NPQ*, which was only 3.0-fold greater than that of the control group, whereas a 5.5-fold increase was observed in Zhoumai 18 (*p* < 0.05) (Figure 2). Moreover, *NPQ* in both wheat cultivars was restored to the control level when the plants were exogenously pretreated with SNP.

**Figure 3:**
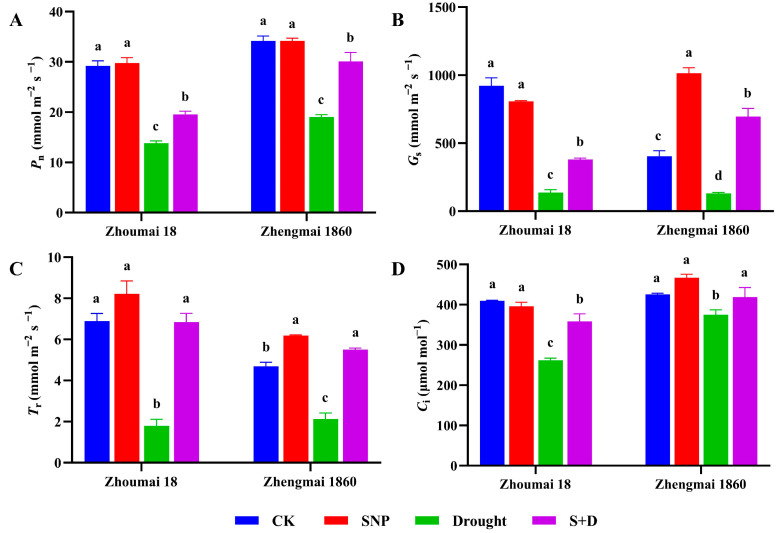
*P*_n_ (**A**), *G*_s_ (**B**), *T*_r_ (**C**), and *C*_i_ (**D**) of Zhengmai 1860 and Zhoumai 18 under drought stress and SNP pretreatment at the grain-filling stage. The wheat plants were cultivated to the grain-filling stage and then subjected to drought stress. The SNP and S+D groups were sprayed with 100 mg/L SNP for 7 days. The watering of the plants in the drought and S+D groups was subsequently stopped to simulate drought stress, whereas those in the CK and SNP groups were watered normally. The data are presented as the means ± standard deviations of three replicates. Different letters indicate significant differences between all treatments at the level of *p* < 0.05.

Photosynthetic rate value (*P*_n_) is an important index to measure the photosynthetic efficiency of plants, and it represents the amount of carbon dioxide absorbed by unit leaf area in a unit time. Stomatal conductance (*G*_s_) is defined as the degree to which the stomata of a plant’s leaves are open to gas. Transpiration rate (*T*_r_) is the amount of water vapor released per unit of leaf area per unit of time. Intercellular CO_2_ concentration (*C*_i_) refers to the CO_2_ concentration in mesophyll intercellular space and is an important parameter to measure photosynthetic efficiency. The photosynthetic characteristics, including the *P*_n_, *G*_s_, *T*_r_, and *C*_i_, of both wheat cultivars significantly decreased in response to drought stress during grain filling (*p* < 0.05) (Figure 3). Compared to the control, Zhoumai 18 exhibited more pronounced reductions in *P*_n_, *G*_s_, *T*_r_, and *C*_i_, with decreases of 53%, 85%, 74%, and 36%, respectively. In contrast, the corresponding reductions in Zhengmai 1860 were comparatively milder, at 44%, 68%, 55%, and 12%, respectively. When exogenous SNP was exogenously applied before drought exposure, the photosynthetic characteristics were significantly restored (*p* < 0.05). In Zhengzhou 1860, *G*_s_, *T*_r_, and *C*_i_ were restored to the level of the CK, and *P*_n_ was restored to 88% of the control value. However, in SNP-pretreated Zhoumai 18, the *P*_n_, *G*_s_, and *C*_i_ under drought stress were still 33%, 59%, and 13% lower, respectively, than those in the corresponding normal watered controls (*p* < 0.05). These results indicate that exogenous SNP pretreatment helps maintain the photosynthetic characteristics of grain-filling wheat plants under drought stress.

**Figure 4:**
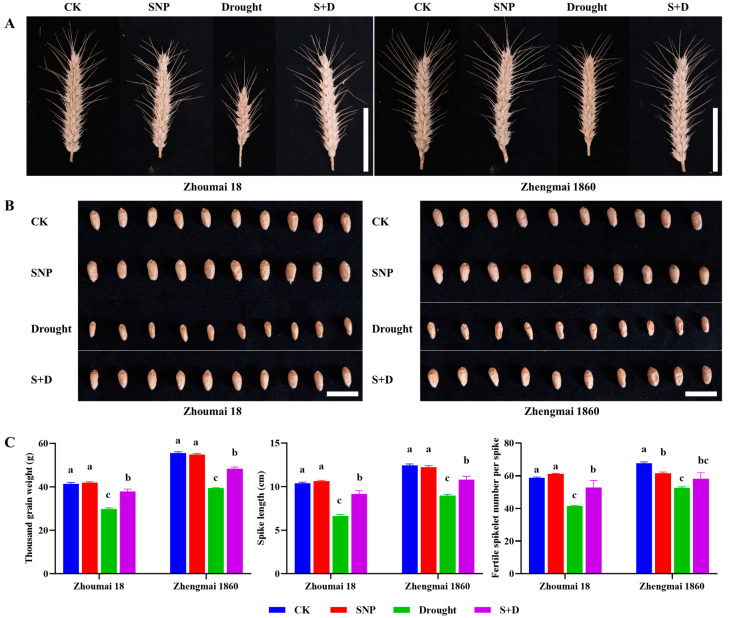
Spike (**A**) and grain (**B**) phenotypes, and yield traits (**C**) of Zhengmai 1860 and Zhoumai 18 under drought stress and SNP pretreatment at the grain-filling stage. The wheat plants were cultivated to the grain-filling stage and then subjected to drought stress. The SNP and S+D groups were sprayed with 100 mg/L SNP for 7 days. The watering of the plants in the drought and S+D groups was subsequently stopped to simulate drought stress, whereas those in the CK and SNP groups were watered normally. The data are presented as the means ± standard deviations of three replicates. Different letters indicate significant differences between all treatments at the level of *p* < 0.05. Scale bars (**A**,**B**), 5 cm for spikes, and 1 cm for grains.

Under drought stress, the thousand-grain weight of Zhengmai 1860 decreased by approximately 15 g compared to the CK group, with a reduction in spike length of about 4 cm and a decrease of approximately 15 grains per spike. In contrast, the thousand-grain weight of Zhoumai 18 dropped by around 10 g, with a similar reduction in spike length (approximately 4 cm) but a slightly greater decrease in the number of grains per spike (about 17 grains per spike) (*p* < 0.05) (Figure 4). Moreover, the grain surface wrinkled due to the low plumpness, as shown by the phenotype observations. Compared with Zhengmai 1860, drought stress had a more severe negative effect on the yield traits of Zhoumai 18. Compared with those of the drought-treated plants without SNP pretreatment, the yield traits clearly increased when exogenous SNP was applied before drought exposure (*p* < 0.05). However, the yield traits of both cultivars under drought stress were still lower than those under CK (*p* < 0.05).

### 2.2. Regulation of Proline Pathway Gene Transcription in Flag Leaves of Wheat at Filling Stage Under Drought Stress

**Figure 5:**
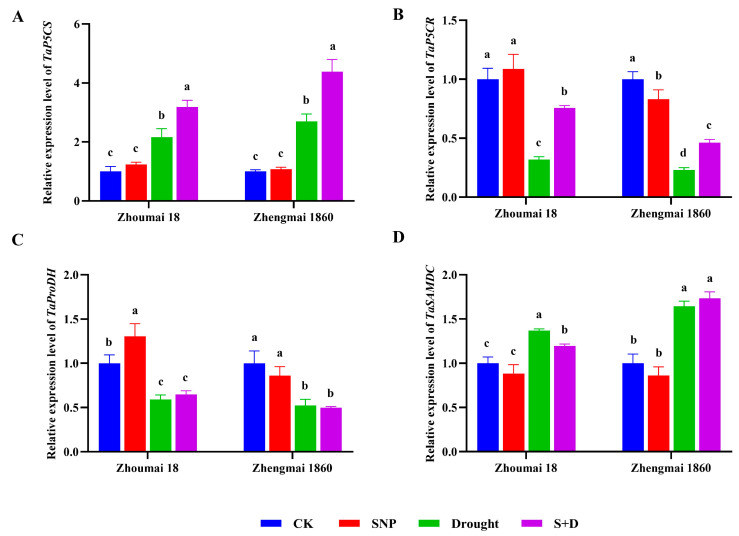
The relative expression levels of *TaP5CS* (**A**) *TaP5CR* (**B**), *TaProDH* (**C**), and *TaSAMDC* (**D**) in different wheat cultivars under drought stress and SNP pretreatment at the grain-filling stage. The wheat plants were cultivated to the grain-filling stage and then subjected to drought stress. The SNP and S+D groups were sprayed with 100 mg/L SNP for 7 days. The watering of the plants in the drought and S+D groups was subsequently stopped to simulate drought stress, whereas those in the CK and SNP groups were watered normally. The data are presented as the means ± standard deviations of three replicates. Different letters indicate significant differences between all treatments at the level of *p* < 0.05.

In contrast, drought stress induced an increase in *TaP5CS* transcription, and a comparatively greater increase was detected in Zhengmai 1860; the increase was about 1.5 times that of Zhoumai 18 (*p* < 0.05) (Figure 5). When exogenous SNP was applied, the induction of the *TaP5CS* gene was further enhanced under drought stress, although SNP had no significant effect under normal watering conditions. Additionally, the improvement effect of SNP was more pronounced in Zhengmai 1860 than in Zhoumai 18. Drought stress significantly decreased the transcription of the *TaP5CR* and *TaProDH* genes, the transcript level of the *TaP5CR* gene in Zhengmai 1860 was reduced to one-fourth of the control group, while that of the *TaProDH* gene decreased to half of the control group. In Zhoumai 18, the transcript level of the *TaP5CR* gene dropped to two-fifths of the control group, whereas the *TaProDH* gene transcript level was reduced to three-fifths of the control group, and *TaSAMDC* transcription was obviously increased in both wheat cultivars under drought stress. The transcript level of the *TaSAMDC* gene in Zhengmai 1860 was 1.6 times that of the control group, while in Zhoumai 18, it was 1.4 times that of the control group (*p* < 0.05) (Figure 5). The drought-induced decrease in *TaP5CR* transcription was reversed by the exogenous preapplication of SNP, which was more significant in Zhoumai 18. Compared with the control group, the recovery degree was 1.65 times that of Zhengmai 1860. However, SNP pretreatment before drought exposure had no significant influence on the transcription of *TaProDH* in either the wheat cultivar or *TaSAMDC* in Zhengmai 1860. Moreover, *TaSAMDC* transcription was obviously inhibited by SNP (*p* < 0.05).

## 3. Discussion

Crop plants can cope with the destructive effects of drought stress through different adaptation mechanisms, such as the use of exogenous substances, transcription factors, and molecular chaperones to resist drought stress and increase plant resistance. Studies have shown that under cold stress, receptor proteins sense the stress and trigger signal transduction, which activates and regulates the expression of CBF expression inducers (ICE) [33]. *TaBiPs*, as molecular chaperones, are significantly upregulated during seedling growth and grain development under drought stress, indicating that they play a crucial role in protecting plants against abiotic stress [34]. In addition, different exogenous substances can also improve the resilience of plants. Research has demonstrated that exogenous application of BR effectively mitigates oxidative and osmotic stress in tea plants subjected to salt stress [35]. Further research has revealed that exogenous ABA treatment in Astragalus activates the stress resistance system of Candida albicans. The central gene *4CL1* (4-coumarate-CoA ligase 1) is translated and expressed in yeast, enhancing the salt tolerance of the transgenic yeast. This, in turn, contributes to increased salt tolerance in plants [36]. Previous studies have shown that SNP can alleviate drought stress damage in sunflowers [37]. In rice, the combined application of exogenous SNP and sodium hydrosulfide effectively relieves the toxicity caused by chromium [38]. An existing study showed that exogenous SNP could augment drought stress tolerance in wheat during the grain-filling stage by increasing psbA transcription [39].

In this study, SNP effectively relieved membrane lipid peroxidation damage in wheat and thus improved the drought tolerance of wheat, which was associated with improving the function of the hydrogen peroxide-degrading enzyme APX by reducing the concentration of oxygen free radicals and relieving oxidative damage to cells [40]. Interestingly, drought alleviation by exogenous SNP was more apparent in the drought-resistant cultivar Zhengmai 1860, as shown by the visual images, SPAD values, MDA contents, and APX activity. These results suggest a possible contribution of cultivar-dependent drought tolerance to the regulatory capacity of exogenous SNP.

Some studies have shown that under drought stress, *T*_r_ and *C*_i_ are weakened to different degrees [41]. The *C*_i_ of wheat flag leaves decreases in response to drought stress, which affects the carboxylation rate in the dark reaction [42]. Compared with that of Zhoumai 18, the photosynthetic capacity of Zhengmai 1860 was damaged to a lesser degree, which should contribute to the greater capacity for yield maintenance when grain-filling plants are subjected to drought stress. When the plants were pretreated with SNP prior to drought stress, both the *P*_n_ and *G*s significantly increased. Compared with Zhoumai 18, Zhengmai 1860 presented a more significant increase in *P*_n_ (Figure 3), indicating that the use of exogenous SNP can increase stomatal conductance [43]. An increase in *NPQ* is a quick response under stress conditions, which prevents the formation of excessive ROS by dissipating more light energy [44]. In this study, the *NPQ* of the two wheat cultivars increased significantly under drought stress. Compared with the other cultivar, Zhengmai 1860 presented a lower increase; it can consume more light energy, avoid the formation of excessive ROS, reduce oxidative damage, and thus maintain the stability of the cell membrane by alleviating the damage caused by drought stress.

Proline is one of the most important organic osmoregulatory substances in plants. As an osmoprotectant that stabilizes cell membranes, proline improves plants’ osmotic regulation ability, maintains the water content in plants, and reduces the water supply for plants under drought stress [45]. Proline can also improve the activity of antioxidant enzymes, remove excess free radicals in plants, and maintain the stability of cell membranes [14]. Drought stress increased the APX content in wheat flag leaves during the grain-filling stage, thereby inhibiting the massive accumulation of H_2_O_2_, preventing membrane lipid peroxidation, reducing oxidative damage in cells, and mitigating the damage caused by drought stress [46]. The SNP pretreatment increased the accumulation of proline and the osmotic regulation ability of wheat leaves while increasing the activity of the APX enzyme and preventing membrane lipid peroxidation in wheat flag leaves. Compared with Zhoumai 18, Zhengmai 1860 accumulated more proline under drought stress, indicating that Zhengmai 1860 has greater osmotic regulation ability.

The synthesis and degradation of proline are carried out mainly by pyrroline-5-carboxylate synthase (P5CS) and proline dehydrogenase (ProDH), respectively [47]. In the cytoplasm, pyrroline-5-carboxylate reductase (P5CR) is an intermediate metabolite of proline and arginine and can reduce pyrroline-5-carboxylate (P5C) to proline [48]. S-Adenosylmethionine decarboxylase (SAMDC) synthesizes decarboxylated S-adenosine methionine (dcSAM) from SAM and is considered the key enzyme for synthesizing Spd and Spm [49]. Under drought stress, *TaP5CS* and *TaSAMDC* transcription increase significantly to promote proline synthesis, whereas *TaProDH* transcription decreases significantly to reduce proline degradation, which synthetically contributes to the accumulation of proline [50]. Exogenous SNP pretreatment promoted the transcriptional recovery of *TaP5CR* and further enhanced *TaP5CS* transcription, indicating that exogenous SNP can improve the osmotic regulation ability of wheat under drought stress [51]. However, a similar effect was not observed for the *TaProDH* and *TaSAMDC* genes. Compared with that in Zhoumai 18, *TaP5CS* gene transcription was more significantly induced in Zhengmai 1860 by drought stress and exogenous SNP pretreatment. This trend coincided with the proline content, indicating that the *TaP5CS* gene plays an important role in proline accumulation in response to drought stress and SNP pretreatment. The excessive accumulation of proline facilitates the maintenance of redox balance and reduces the harmful effects of excess ROS, thus alleviating the damage to wheat caused by drought stress [52].

When the drought-treated wheat plants during the grain-filling stage were cultivated until harvest, significant decreases in thousand kernel weight, ear length, and effective grain number per ear were detected. Compared with Zhoumai 18, Zhengmai 1860 can better maintain yield traits under drought stress and exogenous SNP pretreatment, which should be attributed to Zhengmai 1860, causing less damage to the photosynthetic capacity of flag leaves, thereby reducing dry matter accumulation. Proline can stably maintain pollen fertility and limit seed loss under salt stress, thereby increasing the number of grains per spike in crops [53]. The combined application of proline and salicylic acid (SA) could improve rice biomass and yield by increasing osmoprotectants, improving nutrient transport, increasing antioxidant enzyme activity, and inhibiting oxidative stress [54]. This study demonstrates that drought stress increases proline accumulation by regulating *TaP5CS* transcription. Additionally, exogenous SNP pretreatment enhanced this regulatory effect, further supporting the maintenance of yield traits.

**Figure 6:**
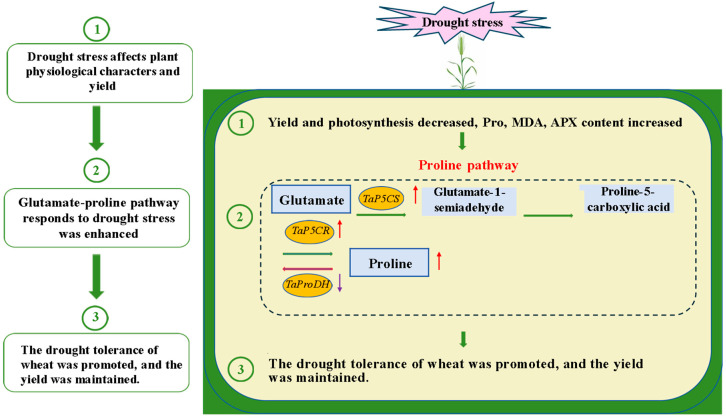
Role of the wheat proline synthesis pathway in response to drought stress and exogenous SNP pretreatment. Red arrows indicate an increase in the expression of related gene transcripts, and purple arrows indicate a decrease in the expression of related gene transcripts.

In summary, during the grain-filling stage under drought stress, the drought-tolerant wheat cultivars Zhoumai 18 and Zhengmai 1860 exhibited different responses. Zhengmai 1860 was less affected by the adverse effects of drought stress and responded more effectively to exogenous SNP pretreatment (Figure 6). Additionally, Zhengmai 1860 demonstrated a greater accumulation of osmotic substances compared to Zhoumai 18. Drought stress upregulated the expression of the *TaP5CS* gene, with Zhengmai 1860 showing a more pronounced increase. These transcriptional changes facilitated the synthesis and accumulation of proline. In the drought-stressed group, SNP pretreatment further enhanced proline accumulation, increased the net photosynthetic rate, and ultimately promoted dry matter accumulation and improved yield traits.

## 4. Materials and Methods

### 4.1. Wheat Plant Materials, Growth Conditions, SNP Pretreatment, and Drought Stress Treatment

The wheat cultivars used in this experiment were Zhoumai 18 and Zhengmai 1860, both of which were provided by the Wheat Research Institute of Henan Academy of Agricultural Sciences. Seeds with plump and uniform grains were used for the experiment. The seeds were sterilized with 5% H_2_O_2_ for 5 min, washed 8–10 times with sterile water, and then soaked in distilled water in a 16 × 16 cm Petri dish. The seeds were incubated in the dark at 24 °C for 12 h until the coleoptiles were obvious, and the swollen seeds were placed on a plate covered with two layers of filter papers. The coleoptiles were placed in the same direction, and the seeds were evenly spread in a constant-temperature incubator at 25 °C. After the wheat seeds were cultured for 3 days and germinated to a depth of 1 cm, seedlings with consistent growth were selected and transplanted into plastic pots (46 cm in length, 34 cm in width, and 15 cm in height) with holes at the bottom. Each plot was filled with 6 kg of soil (nutrient:vermiculite = 3:1), the soil nitrogen content was 51 mg/kg, the phosphorus content was 140 mg/kg, and the potassium content was 240 mg/kg. Each cultivar of wheat was planted in 24 pots with 30 plants in each pot in an artificial climate room under set environmental conditions [14]. Conventional field water, fertilizer, weeding, and insect control management were performed during the plant growth period. Each cultivar of wheat was randomly divided into 4 groups, and each group had 6 small pots and was regarded as one replicate; that is, each treatment was repeated 6 times. Before the drought treatment during the grain-filling stage, the SNP and S+D groups were sprayed with 100 mg·L^−1^ SNP for 7 days. The watering of the plants in the drought and D+S groups was subsequently stopped to simulate drought stress, whereas those in the CK and SNP groups were watered normally.

### 4.2. Measurement of the MDA Content and SPAD

Approximately 1.0 g of wheat flag leaf was ground and homogenized by adding 2 mL of 10% trichloroacetic acid (TCA) solution. The homogenate was then transferred to a clean centrifuge tube. The MDA content was determined via colorimetry [55].

At 10:00 a.m., the chlorophyll content of the center of the flag leaf was measured via a SPAD 502 PLUS (Konica Minolta, Tokyo, Japan) chlorophyll analyzer, according to the manufacturer’s recommendation. Wheat leaves with the same phenotype were selected for each cultivar and each treatment. The samples in each treatment were measured 20 times.

### 4.3. Determination of Ascorbate Peroxidase (APX) Activity

The APX activity was determined as described by Prochazkova et al. [56]. One gram of fresh weight flag leaves was excised and ground with a pestle in an ice-cold mortar with 8 mL of 50 mM phosphate buffer (pH 7.0) containing 0.1 mM EDTA, 5 mM ascorbate, 0.5% (*m*/*v*) PVP, 0.1% (*v*/*v*) Triton X-100, and 0.05% (*v*/*v*) b-mercaptoethanol. The homogenates were filtered through four layers of gauze and then centrifuged at 12,000× *g* for 10 min at 4 °C. The APX activities of the supernatants were assayed by measuring the absorbance at 290 nm via a microplate reader (SpectraMax-i3x, Molecular Devices, Sunnyvale, CA, USA).

### 4.4. Determination of Chlorophyll Fluorescence Parameters

In the morning at 9:00~10:00, the chlorophyll fluorescence parameter was measured via the portable chlorophyll fluorescence instrument MINI-PAM-II (WALZ, Nürnberg, Germany), according to the manufacturer’s recommendation. The leaves were dark-adapted for 20 min and measured under an actinic light intensity of 600 µmol·m^−2^·s^−1^ and a saturation flash intensity of 8000 µmol·m^−2^·s^−1^. The *F_v_*/*F_m_*, *Y*_(II)_, *ETR*, *qP*, and *NPQ* values were calculated according to Kooten and Snel [57].

### 4.5. Determination of Photosynthetic Gas Exchange Parameters

The photosynthetic gas exchange parameters of the flag leaves were determined at 9:00~10:00 via a Li-6400 portable photosynthesis assay system (LI-COR, Lincoln, NE, USA). Three randomly selected plants were used for evaluating various parameters, including the net photosynthetic rate (*P*_n_), transpiration rate (*T*_r_), stomatal conductance (*G*_s_), and intercellular CO_2_ concentration (*C*_i_). The light source was set at 1000 µmol m^−2^ s^−1^ of PPFD with ambient air temperature and humidity, and the reference CO_2_ was set at 365 ppm. The leaf water use efficiency (WUE) was calculated according to the *P*_n_ and *T*_r_ (*P*_n_/*T*_r_) [58].

### 4.6. Determination of Proline Content

The proline contents of leaves in wheat were estimated using the methodology proposed by Bates et al. [59]. Approximately 0.1 g of leaves were homogenized in 1 mL of precooled 3% sulfosalicylic acid for proline extraction. The proline contents were quantified via a standard curve based on the light absorption values measured at 520 nm using a microplate reader (SpectraMax-i3x, Molecular Devices, Sunnyvale, CA, USA).

### 4.7. Reverse Transcription Real-Time PCR Analysis

Total wheat RNA was extracted via an OminiPlant RNA Kit (DNase I) (CWBIO, Taizhou, China). The cDNA was synthesized via a HiScript^®^ II Q RT SuperMix for qPCR (+gDNA wiper) reverse transcription kit (Vazyme, Nanjing, China). Real-time PCR was performed via a 2×ChamQ Universal SYBR qPCR Master Mix Kit (Vazyme, Nanjing, China) and a Bio-Rad iQTM5 Real-Time Fluorescent Quantitative PCR instrument (Bio-Rad Labor-atories Co., Ltd., Hercules, CA, USA), according to the manufacturer’s recommendations. The primers used were designed with Primer 5 (Premier, Inc., Charlotte, NC, USA) and synthesized by Shangya Biotechnology Co., Ltd. (Shanghai, China) (Table 1). Three biological replicates were performed for each treatment. *β*-*Actin* was used as the internal reference gene, and the relative expression of each gene was calculated via the 2^−ΔΔCt^ method [60].

### 4.8. Wheat Yield Trait Analysis

A total of three yield traits, namely, thousand-grain weight, spike length, and fertile spikelet number per spike, were evaluated at the maturity stage. The spike length and fertile spikelet number per spike were determined for 10 randomly selected spikes from each treatment and wheat cultivar. The seeds were thoroughly cleaned, and all non-wheat materials and broken kernels were removed before thousand-grain weight evaluation. Thousand-grain weight was measured in grams by weighing three groups of 1000 grains from each treatment and cultivar.

### 4.9. Statistical Analysis

All the treatments for each cultivar were repeated three times, and the results were calculated and expressed as the mean ± standard deviation. The data were statistically analyzed via Origin2022 software (Originlab, Northampton, MA, USA). The significance of differences between different treatments was compared via one-way repeated-measures ANOVA and a Sidak multiple comparison post hoc test at a level of *p* < 0.05.

## Figures and Tables

**Table 1:** The primer sets used in this study.

Primer Name	Sequnce (5′-3′)	Purpose	Reference
Actin-F	TGCTATCCTTCGTTTGGACCTT	Internal reference	[61]
Actin-R	AGCGGTTGTTGTGAGGGAGT		
TaP5CS-F	GTCCCGACCTGATGCCTT	RT–qPCR	Own design
TaP5CS-R	GGAATCCTTACCACGCCA		
TaP5CR-F	TGTTCAATCGTCAGCCTCCG	RT–qPCR	Own design
TaP5CR-R	GCGAGGGCGTTTTAGGAGTA		
TaProDH-F	GCGACGGAGTTAGGAGTTGT	RT–qPCR	Own design
TaProDH-R	AGGTGTCTGGTCCTTTCCGT		
TaSAMDC-F	TCTGGCGGCAATGCTTATGT	RT–qPCR	Own design
TaSAMDC-R	CGCAGGAAACGTGGCTATCA		

## Data Availability

Data will be made available on request.
